# A missed diagnosis of acute aortic syndrome is associated with ischaemic ECG changes and an initial suspicion of myocardial infarction: a retrospective observational study

**DOI:** 10.1186/s12873-025-01404-8

**Published:** 2025-11-28

**Authors:** Hannah Schönbeck, Anders Björkelund, Emilie Schønbeck Møller, Ulf Ekelund, Jonas Björk, Jakob Lundager Forberg

**Affiliations:** 1 Department of Emergency Medicine, Helsingborg Hospital, Helsingborg, Sweden; 2 Centre for Environmental and Climate Science, Lund University, Lund, Sweden; 3 Department of Emergency Medicine, Bispebjerg Hospital, Copenhagen, Denmark; 4 Department of Internal and Emergency Medicine, Skåne University Hospital, Lund, Sweden; 5 Department of Clinical Sciences, Lund University, Lund, Sweden; 6 Department of Laboratory Medicine, Lund University, Lund, Sweden; 7 Clinical Studies Sweden, Forum South, Skåne University Hospital, Lund, Sweden

**Keywords:** Acute aortic syndrome, Acute aortic dissection, Missed diagnosis, Diagnostic delay

## Abstract

**Background:**

Acute aortic syndrome (AAS) is a life-threatening condition reported as often misdiagnosed. Advancements in CT availability and diagnostic algorithms, including high-sensitivity troponin and D-dimer for acute coronary syndrome (ACS) and pulmonary embolism (PE), may reduce AAS misdiagnosis, but the impact remains unclear. This study evaluated the incidence, characteristics, and clinical features of missed AAS diagnoses in Swedish emergency departments (EDs) compared with those identified during initial ED visit.

**Methods:**

A retrospective observational study was conducted on 630,275 ED visits in Region Skåne, Sweden, from 2017 to 2018. Cases of AAS diagnosed within 30 days were identified via national registers. Clinical features, imaging, ECG findings, blood tests, outcomes, and time of diagnosis were reviewed. Missed diagnosis was defined as AAS not identified in the ED, with the patient being either discharged or admitted without imaging to detect AAS. Aortic dissection detection risk score (ADD-RS) combined with D-dimer levels were also retrospectively calculated.

**Results:**

Among 98 confirmed AAS cases, 82 were diagnosed during the initial ED visit, whereas 16 (16%; 95% CI: 9.6–25.1%) were initially missed, with 2 discharged from the ED. Patients with missed AAS diagnoses were more likely to present with ST elevation/depression than were those diagnosed during the initial ED visit (43% vs. 15%, *p* = 0.01). ACS was the initial working diagnosis in 7 of 16 of the missed cases. The median time to diagnosis was significantly longer in missed cases (19 h [2–192] vs. 2 h [0.5–11.6], *p* < 0.001). Missed cases had higher 30-day (25% vs. 16%) and 90-day mortality rates (38% vs. 18%), although these differences were not statistically significant. In 55 of the 59 AAS patients, the ADD-RS plus D-dimer algorithm recommended CT of the aorta (93% sensitivity).

**Conclusion:**

Missed AAS remains a diagnostic challenge, with a miss rate of approximately one in six in Swedish EDs. Patients with missed AAS are more likely to present with ST changes and an initial suspicion of ACS. Given the overlap of symptoms, such as chest pain, with other critical conditions like ACS and PE, future ED diagnostic tools should be developed to predict multiple critical diagnoses concurrently.

## Background

Acute aortic syndrome (AAS), which includes acute aortic dissection, intramural hematoma, and penetrating aortic ulcer, is a rare but life-threatening emergency that requires prompt recognition and management in the emergency department (ED) [1, 2]. Although uncommon, its clinical importance stems from the severe consequences of delayed diagnosis, with mortality rates rising by 1–2% per hour without intervention [3]. The diagnostic challenge in the ED is amplified by the symptom overlap (e.g., chest pain) between the AAS and more common conditions, such as acute coronary syndrome (ACS) and pulmonary embolism (PE) [4, 5].

Misdiagnoses of AAS have been reported to be prevalent, with rates of up to 39% [4]. Misdiagnosed cases often present with atypical symptoms or mimic other conditions, leading to inappropriate treatments, such as anticoagulation, and delayed diagnoses [4, 5]. Advances in the availability of computed tomography (CT) imaging have improved diagnostic capabilities and reduced the threshold for advanced imaging in many healthcare systems. CT, the gold standard for diagnosing AAS, is now more accessible, theoretically decreasing the number of missed cases.

Chest pain is the most common symptom of AAS, yet it is also a hallmark symptom of other conditions, such as ACS and PE, which complicates the diagnostic process [5, 6]. The early diagnosis of ACS and PE has benefitted from numerous well-studied decision-support tools and algorithms. However, the development of similar tools for AAS has been more challenging because of AAS low prevalence and diverse presentation. Nonetheless, decision-support models, such as the aortic dissection detection risk score (ADD-RS) combined with biomarkers such as D-dimer, have shown promise in enhancing diagnostic accuracy and reducing unnecessary imaging in the diagnosis of aortic dissection [7–9]. Despite these advancements, elevated biomarkers such as troponin and D-dimer, along with electrocardiographic findings, can mislead clinicians toward the ACS or PE pathways instead of the AAS [4, 5, 10], delaying its diagnosis.

With the increasing use of high-sensitivity troponin and ACS decision algorithms, it remains unclear how these advances in ACS and PE diagnostics have influenced the incidence of missed AAS cases. The focus and implementation of diagnostic algorithms for one condition are rarely studied in the context of related diagnoses such as AAS [5].

The primary aim of this study was to assess the incidence, characteristics, and clinical features of AAS in patients presenting to the ED, comparing those with timely diagnoses to those initially missed. A secondary aim was to evaluate whether the application of the ADD-RS and D-dimer test could reliably identify AAS cases in our Swedish healthcare setting.

### Methods

This retrospective observational study included all patients presenting to the eight emergency departments (EDs) in Region Skåne, Sweden, from 2017 to 2018. Data were obtained from the Skåne Emergency Medicine (SEM) Cohort, an integrated database that combines clinical, diagnostic, and demographic information with data from regional and national registers. Region Skåne serves a population of approximately 1.3 million inhabitants.

The SEM cohort was used to identify all patients diagnosed with AAS (ICD 171.0), including those with aortic dissection, intramural hematoma, and penetrating atherosclerotic ulcer, within 30 days of the index emergency department visit. Patients who died within 30 days of the index ED visit with a recorded cause of death attributed to AAS (including autopsy findings) were also included. Data on previous medical history, redeemed prescriptions, ED and imaging timestamps, imaging findings, and blood test results were collected for analysis.

A review of medical records from the ED visit and hospital stay as well as the subsequent 30 days was conducted for all patients with a recorded diagnosis of aortic syndrome as described above, including those with a missed diagnosis of aortic syndrome. A missed diagnosis of AAS was defined as an AAS not identified in the ED, with the patient being either discharged or admitted without imaging to detect AAS. Patients whose review confirmed an AAS diagnosis related to a previous episode were excluded from further analysis. Imaging reports and timestamps were manually examined for accuracy.

Furthermore, the Aortic Dissection Detection Risk Score (ADD-RS) was retrospectively calculated using the documentation available in the medical records. The ADD-RS score, as described by Rogers et al. [11]. , was calculated by assigning one point for the presence of any of three risk categories: high-risk conditions, high-risk pain features, or high-risk examination findings. In summary, high-risk conditions included a history of aortic dissection, thoracic aneurysm, aortic valve disease, or connective tissue disorders. High-risk pain features refer to sudden, severe, or tearing chest, back, or abdominal pain, whereas high-risk examination findings include perfusion deficits, a new diastolic murmur, or hypotension. The score ranged from 0 to 3, with a score of 0 or 1 indicating a low risk where acute aortic dissection could have been ruled out if the D-dimer test was negative, whereas a positive D-dimer test or a score of 2 or higher suggested a higher risk, suggesting a CT aorta for further evaluation. Patients were excluded from the ADD-RS analysis if medical records lacked sufficient information to calculate the score or if D-dimer was missing for scores of 0 or 1. The D-dimer assay used in the study was the Medirox assay (Nordic Biomarker, Umeå, Sweden), with a decision limit (negative test) < 0.25 mg/L.

The time of AAS diagnosis was defined as the time when the CT scan confirmed the presence of an AAS.

In all patients, the ECG was interpreted by an emergency physician (HS). The European Society of Cardiology’s definition of ST elevation was applied [12], and ST depression was defined as > 1 mm in two contiguous leads.

### Statistics

Proportions are reported as percentages with 95% confidence intervals. Descriptive data are reported as the means (with standard deviations) or medians (with 25th and 75th percentiles), as appropriate. The presence of significant differences in case fatality was tested via the log-rank Mantel‒Cox test, whereas the prevalence between groups was assessed via either a chi-square test or Fisher’s exact test, as appropriate. For continuous variables, the Mann–Whitney U test was used when applicable. A two-sided p value of < 0.05 was considered statistically significant. Analysis was performed via IBM SPSS Statistics, version 30.0.0.0 for Windows.

### Ethics

The study was approved by the Swedish Ethical Review Authority (Dnr 2019–05783) and by Region Skåne (302 − 19).

## Results

**Fig. 1:**
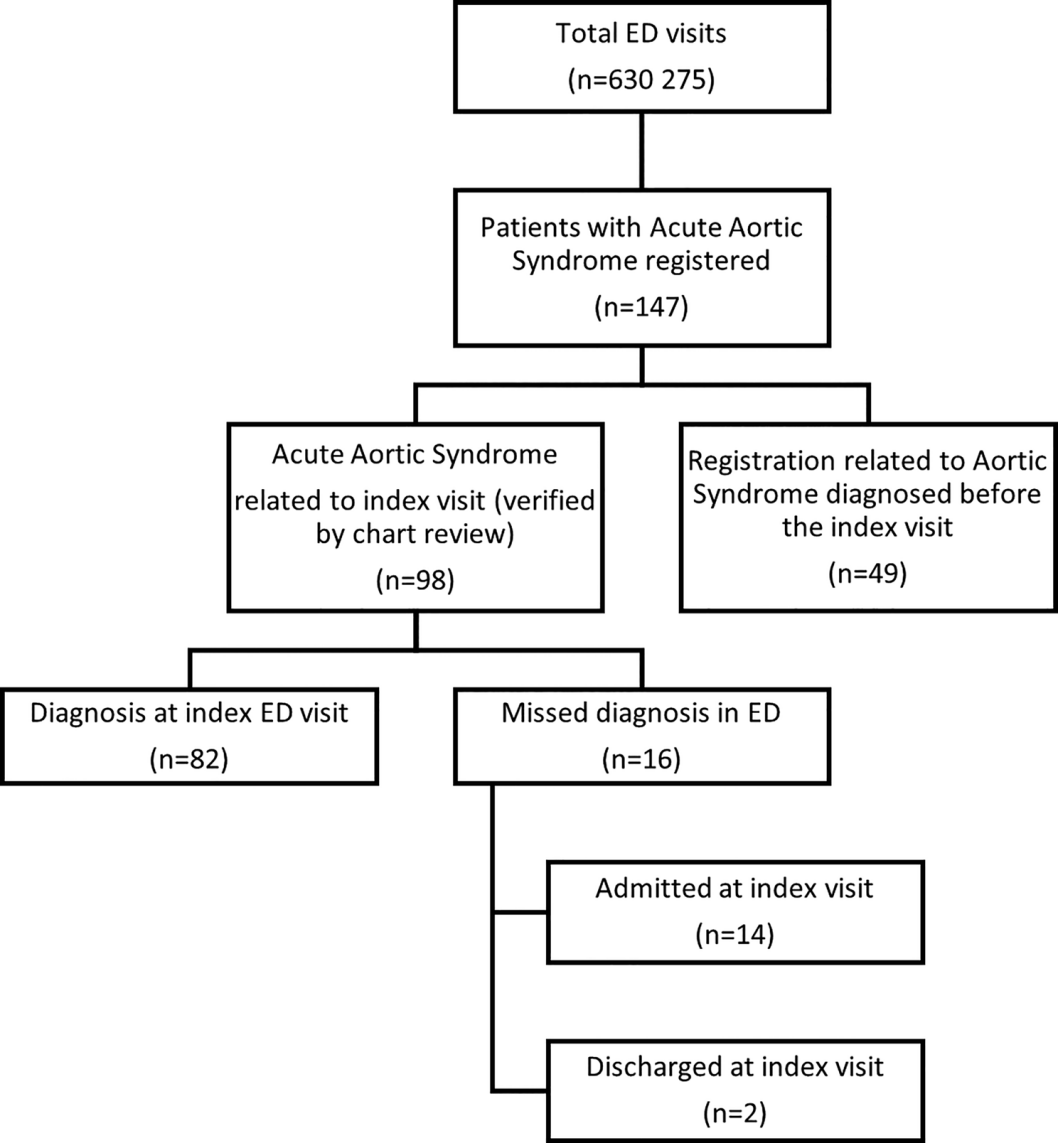
Patient inclusion and disposition. ED: Emergency Department

Among all 630,275 visits across eight EDs, 147 patients were recorded with an ICD code for AAS. Following a review of medical records, 49 of these cases were found to be related to a previous hospital admission and did not represent the initial presentation of AAS (Fig. 1). The remaining 98 cases, in which the initial ED presentation was due to AAS, were included in the study. Of these, 82 cases (83.7%) were correctly identified at the ED index visit, whereas 16 cases (16.3%) were initially missed.

Overall, 62 patients (63%) with AAS were men, and the median age of all AAS patients was 72 (63–78) years. Chest pain was the most common complaint reported by 54 patients (55%). A total of 54 patients (55%) arrived by ambulance. Most patients with AAS presented during the day shift (51 patients, 52%), followed by 27 patients (28%) in the evening shift and 20 patients (20%) during the night shift.

Among the AAS patients, 80 (82%) had dissections, of which 61 were Stanford type A dissections; 15 patients (15%) had intramural hematomas, and 3 (3%) had penetrating atherosclerotic ulcers. A total of 17 patients died within 30 days. Table 1 summarizes the characteristics and outcomes of patients with an AAS diagnosis made at the initial ED visit compared with those in whom the diagnosis was initially missed.

**Table 1:** Characteristics of patients with acute aortic syndrome diagnosed at the ED index visit versus the initial missed diagnosis

	Diagnosed in ED (*n* = 82)	Missed Diagnosis (*n* = 16)
Age, years (median, Q1–Q3)	72 (63.5–77.8)	74 (62–80.8)
Gender (Male)	52 (63%)	10 (63%)
**Type of Aortic syndrome**		
Dissection	69 (84%)	11 (69%)
Stanford Type A dissection	51 (62%)	10 (63%)
Intramural hematoma	11 (13%)	4 (25%)
Penetrating ulcer	2 (2.4%)	1 (6.3%)
**Chief presenting complaint**		
Chest pain	46 (56%)	8 (50%)
Back/abdominal pain	16 (20%)	0
Dyspnea	1 (1.2%)	2 (13%)
Neurological deficit	8 (9.8%)	1 (6.3%)
Syncope/unconscious	4 (4.9%)	0
Dizziness	0	2 (13%)
Cardiac arrest	1 (1.2%)	1 (6.3%)
Extremity pain	3 (3.7%)	0
Arrhythmia	2 (2.4%)	0
Other	1 (1.2%)	2 (13%)
**Previous medical history**		
Aortic dissection	6 (7.3%)	0
Hypertension	50 (61%)	12 (75%)
Ischemic heart disease	10 (12%)	1 (6.3%)
Diabetes	5 (6.1%)	2 (13%)
Stroke	6 (7.3%)	1 (6.3%)
COPD	11 (13%)	3 (19%)
Marfan Syndrome	1 (1.2%)	0
Charlson Comorbidity Index (median, Q1–Q3)	1 (1–3)	1 (1–3)
**Medications**		
Insulin	1 (1.2%)	0
Other antidiabetic agents	4 (4.9%)	1 (6.3%)
Anticoagulants	40 (49%)	5 (31%)
Beta-blockers	36 (44%)	6 (38%)
Calcium channel blockers	41 (50%)	8 (50%)
Agents affecting the RAAS	40 (49%)	6 (38%)
Thiazides	6 (7.3%)	1 (6.3%)

The most common presenting complaint was chest pain in both patients diagnosed in the ED and those with a missed diagnosis (56% vs. 50%). Nonspecific symptoms of AAS (other than chest, back, abdominal pain, syncope, or neurological deficits) were more prevalent in the missed AAS group than in the timely diagnosis group, with 43.8% in the missed AAS group and 10.8% in the timely AAS group (*p* = 0.001).

Patients with missed AAD had a significantly greater prevalence of ST elevation or depression than did those diagnosed in the ED, with rates of 42.9% and 15.0%, respectively (*p* = 0.026) (Table 2).

**Table 2:** Clinical findings, primary working diagnosis and outcomes of patients with acute aortic syndrome diagnosed at the ED index visit versus initial missed diagnosis

	Diagnosed in ED (*n* = 82)	Missed Diagnosis (*n* = 16)
**ECG at presentation**		
ST depression/elevation	11 of 81 (13.6%)	7 of 13 (53.8%)*
Negative T-wave	6 of 81 (7.4%)	3 of 13 (23.1%)
Left ventricular hypertrophy	3 of 81 (3.7%)	3 of 13 (23.1%)*
**Blood Tests at ED Index Visit**		
Anaemia **	19 of 82 (23%)	5 of 15 (33%)
Lactate elevated***	36 of 82 (44%)	5 of 14 (36%)
D-dimer positive ****	48 of 52 (92%)	7 of 7 (100%)
High-sensitive Troponin T elevated*****	23 of 70 (33%)	7 of 13 (54%)
**Primary working diagnosis in the ED**		
Aortic dissection	72 (88%)	0
Acute Coronary Syndrome	0	7 (44%)*
Stroke	6 (7.3%)	0
Pulmonary embolism	4 (4.9%)	2 (13%)
Infection	0	3 (18%)
Gastritis	0	1 (6.3%)
Musculoskeletal pain	0	1 (6.3%)
Kidney stone	0	1 (6.3%)
Vasovagal syncope/pulmonary embolism	0	1 (6.3%)
**Mortality**		
30-day mortality	13 of 82 (16%)	4 of 16 (25%)
90-day mortality	15 of 82 (18%)	6 of 16 (38%)
**Time to Diagnosis (median**,** range)**	2 h (27 min–11.5 h)	19 h (105 min–8 days)

* Significant difference in proportion between groups (*p* < 0.05), ** haemoglobin < 117 g/L in females and < 134 g/L in males, *** lactate > 2.2 mmol/L if venous or > 1.6 mmol/L if arterial sample, **** D-dimer (Medirox assay) > 0.24 mg/L. ***** High-sensitivity troponin T above the 99th percentile; >15 ng/L

There were no notable differences in age, sex, comorbidity index, or arrival during out-of-office hours between patients with a timely diagnosis of AAS and those whose diagnosis was missed. Intramural hematoma and penetrating ulcers were more common in the missed diagnosis group than in the timely diagnosis group (31.3%, 95% CI: 14.2–55.6% vs. 15.9%, 95% CI: 9.5–25.3%) although this difference was not statistically significant.

**Fig. 2:**
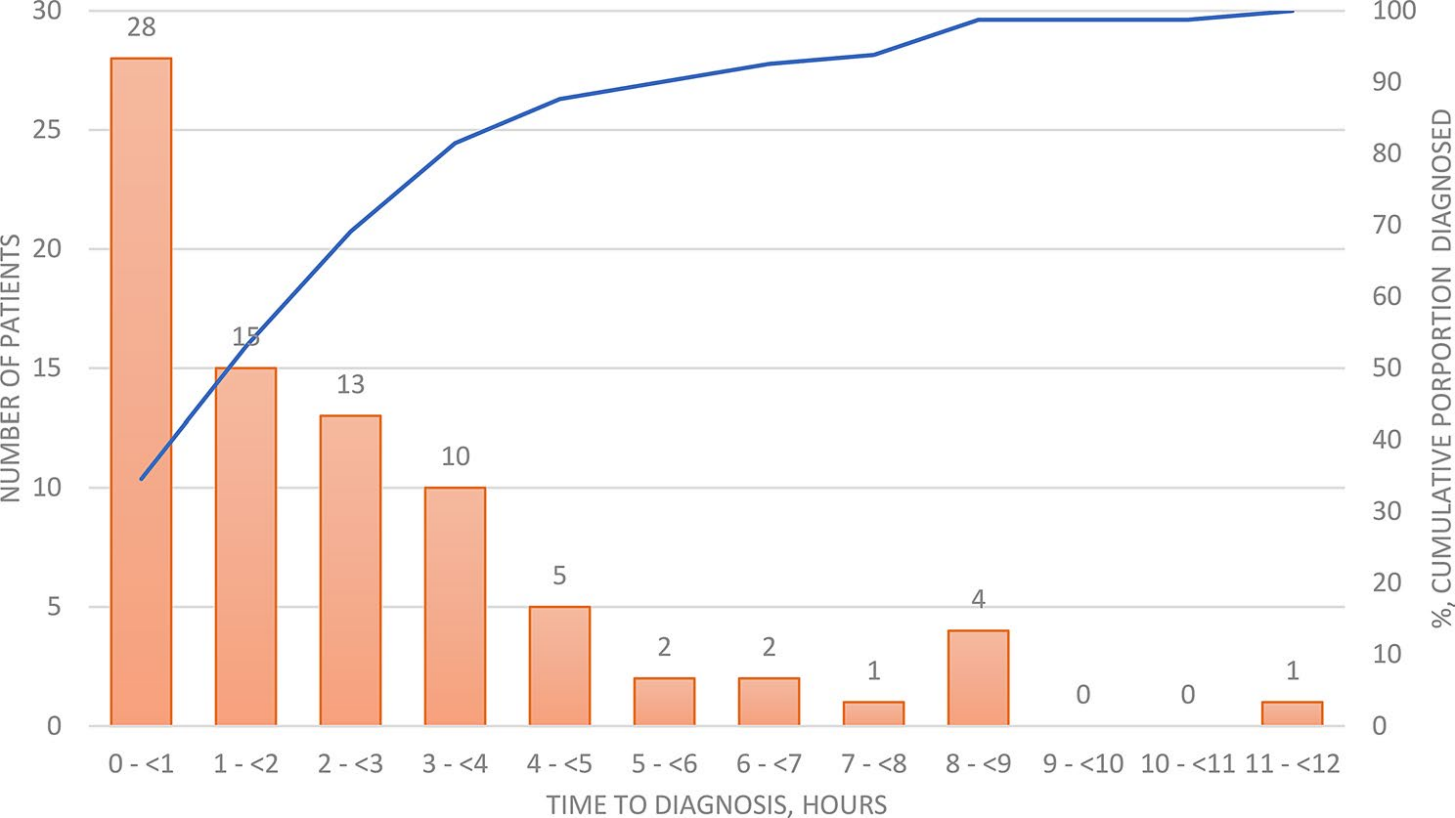
Time to diagnosis of acute aortic syndrome from the time of arrival at the Emergency Department

Patients with an initially missed AAS diagnosis had a higher 90-day mortality (37.8%, 95% CI: 18.5–61.4% vs. 18.3%, 95% CI: 11.4–28.0%) than those diagnosed at the initial ED visit did (see Fig. 2). However, this difference was not statistically significant (*p* = 0.08).

**Fig. 3:**
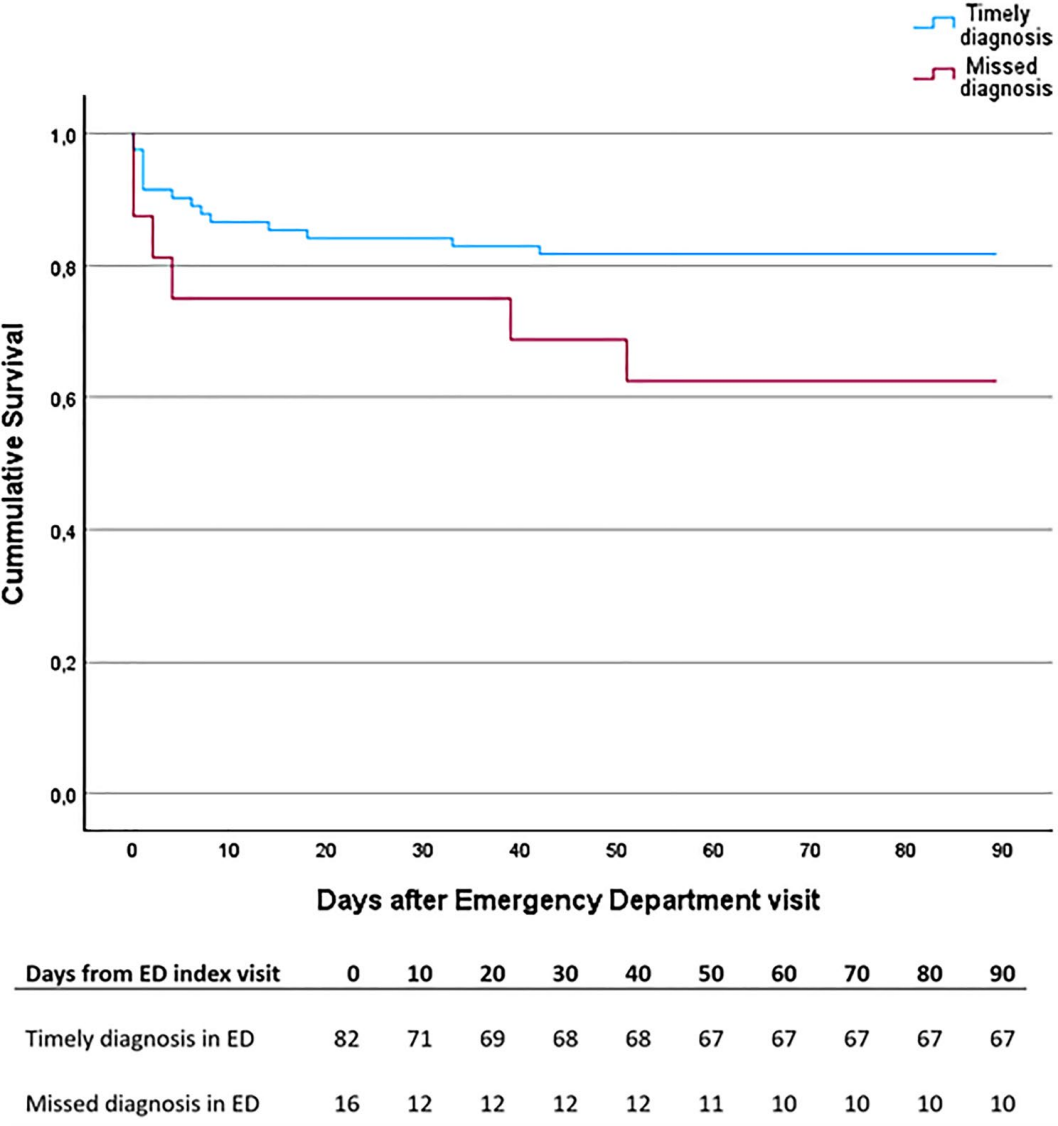
Kaplan–Meier curve comparing survival among patients with acute aortic syndrome who received a timely diagnosis in the emergency department versus those with an initially missed diagnosis. The table below the curve shows the number of patients remaining alive over time following the index emergency department visit

The median time to AAS detection via CT scan was significantly longer in cases where the diagnosis was initially missed, with a median time of 19 h, than in cases where the AAS was promptly identified, with a median time of 2 h. Overall, 28 patients (34%) were diagnosed within the first hour (Fig. 3). The median time to diagnosis was 65 min (Q1–Q3: 54–122) for patients who died within 30 days, whereas it was 120 min (Q1–Q3: 50–220) for those who survived beyond 30 days. However, this difference was not statistically significant (*p* = 0.27).

In cases where the diagnosis was initially missed, the most common primary working diagnosis in the ED was ACS (44%). In 10 of the 82 patients with a diagnosis of AAS in the ED, the primary working diagnosis was not AAS. However, a CT scan ordered from the ED subsequently revealed a dissection. The primary working diagnosis of myocardial infarction was significantly more common in missed AAS patients (7/16) than in patients diagnosed in the ED (1/82) (*p* < 0.0001). Table 3 presents a case discription of missed cases.

**Table 3:** Short case description of missed cases with acute aortic syndrome

Age	Time to diagnosis (hours)	Primary working diagnosis in ED	D-dimer	ADD-RS score	Case description
70+	19	ACS	Positive	1	Chest pain with sudden onset, radiating to the back. History of aortic aneurysm. ECG with unspecific changes. Troponin elevated.
80+	27	PE	Positive	1	Chest discomfort and syncope. CT thorax shows pericardial effusion. Echocardiography reveals dissection. No ischemic ECG changes. Troponin normal.
80+	71	Urosepsis	Not performed	Not assessable	Fall with prolonged time on the floor. CRP 200. No pain complaints in ED. New onset of atrial fibrillation prompted echocardiography, which revealed dissection. ECG with ST-depression No Troponin available.
70+	192	Back pain	Not performed	Not assessable	Lumbal pain. Imaging from primary care two weeks after index visit shows dissection. No ECG or Troponin available.
40+	23	Gastritis	Positive	Not assessable	Chest pain onset while defecating. Epigastric tenderness. Discharged from ED but returned the same day due to pain where ACS is suspected. No ischemic ECG changes. Troponin normal. CT shows dissection.
50+	13	ACS	Not performed	Not assessable	Epigastric pain radiating to the right arm. Suspected ischemic ECG changes. Troponin without changes.
70+	2,5	ACS	Positive	1	Intense chest pain. Minimal ST-elevation initially, coronary angiography normal. Troponin normal. CT shows aortic dissection.
80+	20	Sepsis	Not performed	Not assessable	Strange behavior, calling for help, not answering questions. Tachypnea. No ischemic ECG changes. Troponin elevated.
60+	19	PE	Positive	3	Sudden back pain radiating to the chest. Morphine without effect. Vomiting. No ischemic ECG changes. Elevated Troponin.
40+	9	ACS	Positive	Not assessable	Chest pain, hypotension, and bradycardia. No ischemic ECG changes. Troponin normal. Increased pain six hours later, ECG shows STEMI. Coronary angiography shows dissection. Cardiac arrest before intervention.
50+	2	ACS	Not performed	Not assessable	Sudden chest pain. Suspected STEMI. No ischemic Ecg changes. Troponin elevated. Cardiac arrest with ROSC, coronary angiography reveals dissection.
70+	6,5	ACS	Not performed	Not assessable	Chest pain for three days. Disoriented and hypotensive. No complaints of pain. Ischemic ECG changes. Elevated troponin. Cardiac arrest.
80+	16	ACS	Not performed	Not assessable	Sudden chest pain. AV-block type 2. No ischemic ECG changes. Troponin T elevated. Echocardiography in the ward shows suspected aortic dissection.
80+	84	PE	Positive	1	Sudden dyspnea and abdominal pain, correlated to breathing and movement. No ischemic ECG changes. Troponin elevated.
80+	20	Kidney stone	Not performed	Not assessable	Sudden flank pain. CT scan with suspected dissection. No ischemic ECG changes. Troponin normal.
60+	77	Pneumonia	Not performed	Not assessable	Three days of dyspnea and chest pain correlated with breathing, with sudden onset. CRP 300. Troponin not analysed.

Among the 59 patients with a registered D-dimer test within the first 24 h of initial presentation, 55 had a positive D-dimer (above 0.25 mg/L, no age adjustment was applied), resulting in a theoretical sensitivity of 93.2% (95% CI: 83.5–98.1%) for predicting AAS.

In 59 patients, the documented information and availability of a D-dimer test allowed the calculation of an ADD-RS score (Table 4). In four patients, the ADD-RS score combined with D-dimer measurement would have missed AAS, as the score would have ruled out dissection and not recommended proceeding with a CT scan. Therefore, the estimated sensitivity of the ADD-RS in this study was 93.2% (95% CI: 83.5–98.1%).

**Table 4:** Recommendations if the ADD-RS score would have been applied in the ED. A CT scan was recommended for patients with an ADD-RS score greater than one (high risk) or for those with a score of one or less (low risk) if the D-dimer test was positive. The ADD-RS score could be calculated for 59 out of 98 patients

	CT Recommended	CT Not Recommended
**Final Diagnosis**		
Acute aortic syndrome	55 (93%)	4 (7%)
- Acute aortic dissection	47	3
- Intramural hematoma	7	1
- Penetrating aortic ulcer	1	0

## Discussion

Retrospective analysis of 630,275 ED visits in Region Skåne, Sweden, from 2017 to 2018 identified 98 cases of AAS, of which 16.3% were initially missed in the ED. This is slightly higher, although consistent with the findings of a recent Canadian study by Ohle et al.. (2023), which reported an initial misdiagnosis rate of 12.5% [13]. In contrast, older studies have reported significantly higher rates of missed diagnoses. For example, Hansen et al. (2000–2004) reported a missed AAD rate of 39% in a Canadian population [4], whereas a US study from the 1980s revealed that 28% of AAD cases were only diagnosed postmortem [14]. Although the methodologies used across these studies vary, making direct comparisons challenging, our findings suggest that the missed diagnosis rate in Sweden is comparable to that reported by current North American centers. Furthermore, the reduction in missed diagnoses over the decades likely reflects the increased availability of CT imaging in the ED setting. While there appears to be a trend of decreasing missed diagnoses, the rate remains high, even with a low CT threshold resulting in many negative scans [15], emphasizing the need for improved diagnostic strategies.

Previous studies have identified ACS as the most common misdiagnosis in AAS, which aligns with our findings of 43%. Hansen et al. reported that ACS was frequently misdiagnosed in AAS patients, often leading to inappropriate antithrombotic treatment, which could worsen the prognosis of AAS [4]. Similarly, the IRAD registry highlighted factors associated with delayed recognition and treatment of acute type A aortic dissection, noting that ACS-like symptoms, such as chest pain and ECG changes, were significant contributors [6]. Despite increased CT utilization and newer ACS diagnostic pathways, this issue persists in our study, where ST elevation or depression was significantly more prevalent, occurring in more than half of the patients with a missed AAS diagnosis. Although newer ACS diagnostic pathways incorporate high-sensitivity troponins, our findings revealed no significant difference in the prevalence of elevated high-sensitivity TnT values between the missed and timely diagnosed groups. These findings suggest that elevated TnT is not a major contributor to diagnostic errors leading to the wrong clinical pathway. However, our results indicate that ECG changes may play a prominent role in misdirecting physicians toward an ACS diagnosis. The small size of our study limits definitive conclusions, but it appears that ECG findings are more likely than troponins to misguide clinicians toward the ACS pathway.

The frequent misdiagnosis of AAS as ACS highlights the need for diagnostic algorithms that account for the detection or rule-out of critical conditions with overlapping symptoms, such as chest pain. These algorithms should address not only AAS and ACS but also other critical conditions, such as pulmonary embolism, that share similar clinical presentations [5]. The development of such comprehensive diagnostic tools is particularly challenging for rare conditions such as AAS and will require large, high-quality, multi-informational databases. AI could play a key role in overcoming these challenges by integrating diagnostic information and potentially providing unified decision support for multiple critical conditions.

AAS patients presenting with atypical symptoms, although heterogeneously defined in the literature, pose a significant diagnostic challenge. Atypical presentations have been reported in up to 25% of AAS cases [6] and accounted for approximately 15% in our study. These patients are consistently associated with higher rates of missed diagnoses, delayed recognition and treatment, and increased mortality. Despite this, no clear strategies have been established for reliably identifying these patients beyond heightened clinical awareness and education. Targeted research is urgently needed to develop specific diagnostic approaches for this high-risk subgroup.

In our study, the sensitivity of a positive D-dimer alone was 93% (95% CI: 83.5–98.1%), which overlaps with but is slightly lower than the 96.7% reported in the ADvISED trial [7]. When retrospectively evaluating the diagnostic performance of an ADD-RS score of ≤ 1 combined with a positive D-dimer (> 250 ng/mL), we found a sensitivity of 93% (95% CI: 83.5–98.1%), which is lower than the sensitivity of 98.9–100% reported in meta-analyses [8, 9, 16] and the 98.8% reported in the large prospective multicenter ADvISED study involving 1,850 patients [7]. The retrospective nature of our ADD-RS calculation, where certain score components might not have been documented or assessed by the treating physician, may have impacted sensitivity despite excluding patients with obvious missing data. These findings underscore the importance of thorough data collection for ADD-RS scoring and the need to integrate clinical judgment alongside decision support tools. The ADD-RS was developed to diagnose aortic dissection, not the full spectrum of acute aortic syndromes, and existing evidence supports its use only for diagnosing and ruling out dissections. In our study, three potentially missed cases using ADD-RS and D-dimer were dissections and one was an intramural hematoma (Table 3); excluding the latter raises the sensitivity to 94% (95% CI, 83.8–97.9%).

The strength of this study lies in the SEM cohort, which includes several national registers, ensuring that any documented diagnosis or cause of death related to AAS across Sweden is captured within the dataset. A previous Swedish nationwide population-based study, which included high rates of postmortem examinations, identified 4425 cases of dissection over 15 years in a population of 8.7 million, yielding an annual incidence of 3.4 acute dissections per 100,000 persons [17]. Similarly, the present study revealed an annual incidence of 3.8 AAS per 100,000 inhabitants in our region, which was based on 98 cases over two years in a population of 1.3 million, suggesting that relatively few cases were likely missed.

This study has several limitations. Not all instances of missed AAS in the population were identified, as the study did not include patients who died at home without attending the ED, those who arrived at the ED in cardiac arrest without undergoing autopsy, and cases where the diagnosis was missed in primary care. However, this does not affect cases initially missed in the ED. Theoretically, some patients who died after ED attendance without AAS suspicion could have had undiagnosed AAS, but these cases are likely very few and predominantly among older patients, as unexpected deaths in younger individuals or those with fewer comorbidities would typically undergo autopsy, which would capture AAS as the cause of death. Another limitation is that the specificity of the ADD-RS algorithm could not be assessed, as the retrospective design did not allow identification of true negatives or false positives. It should also be noted that chest CT angiography is the preferred diagnostic modality in Sweden, which may not reflect practice patterns in other countries.

## Conclusion

The miss rate of acute aortic syndrome (AAS) in a modern Swedish emergency department was approximately 16% (95% CI: 9.9%–24.5%). Patients with missed AAS are more likely to present with atypical symptoms, ECG ST changes, and an initial suspicion of ACS. Increased awareness of the characteristics associated with missed AAS could help reduce misdiagnoses. Given the overlap in the clinical presentations of critical conditions such as AAS, ACS, and PE, future decision-support models (e.g., those for patients with acute chest pain) should incorporate the prediction of multiple or all of these conditions.

## Data Availability

Owing to the nature of this retrospective cohort study, data sharing is restricted. The data contain sensitive patient information protected under national privacy regulations and patient confidentiality agreements. As such, the dataset for this study is not publicly available. Access is limited to the research team to maintain patient privacy and adhere to ethical standards. Researchers interested in the study methodology or other nonsensitive materials can contact the corresponding author.

## Abbreviations

AAS: Acute Aortic Syndrome
ACS: Acute coronary syndrome
ADD-RS: Aortic Dissection Detection Risk Score
CT: Computed Tomography
ECG: Electrocardiogram
ED: Emergency Department
ICD: International Classification of Diseases
IRAD: International Registry of Acute Aortic Dissection
PE: Pulmonary embolism
SEM: Skåne Emergency Medicine
ST: ST Segment in Electrocardiogram
TnT: Troponin T
